# Deciphering Mitochondria: Unveiling Their Roles in Mechanosensing and Mechanotransduction

**DOI:** 10.34133/research.0816

**Published:** 2025-08-08

**Authors:** Jiaxuan Yu, Ye Huang, Yujie Qin, Jingfei Zhu, Tian Zhao, Hao Wu, Xi Ye, Xiang Qin, Shun Li, Yungchang Chen, Yiyao Liu, Tingting Li

**Affiliations:** ^1^ Department of Medical Oncology, Sichuan Clinical Research Center for Cancer, Sichuan Cancer Hospital & Institute, Sichuan Cancer Center, and School of Life Science and Technology, University of Electronic Science and Technology of China, Chengdu 610041, Sichuan, P. R. China.; ^2^ TCM Regulating Metabolic Diseases Key Laboratory of Sichuan Province, Hospital of Chengdu University of Traditional Chinese Medicine, Chengdu 610072, Sichuan, P. R. China.; ^3^ Department of Otorhinolaryngology, Hospital of Chengdu University of Traditional Chinese Medicine, Chengdu 610072, P. R. China.; ^4^ Department of Urology, Deyang People’s Hospital, Deyang 618099, Sichuan, P. R. China.

## Abstract

Mitochondria are highly dynamic organelles that are responsible for essential cellular functions such as calcium regulation, reactive oxygen species (ROS) production, metabolism, and apoptosis initiation. Mitochondrial dysfunctions are associated with a variety of pathologies, and the onset and progression of disease are accompanied by alterations in extracellular biochemical and mechanical signals. Recent studies have demonstrated that physicochemical cues, especially mechanical cues, exert pivotal roles in the organization of mitochondrial network and their metabolic functions. Therefore, understanding the mechanisms that orchestrate mitochondrial morphology and function is essential for elucidating their role in both health and disease. This review discusses novel insights into the recent advances regarding mitochondrial dysfunction across a spectrum of diseases and describes the effect of various factors. It then highlights the recently discovered mechanisms, particularly those involving matrix mechanical cues and cellular mechanical cues, summarizing the multiple pathways of mechanotransduction, such as integrin, Piezo1/TRPV4, and YAP/TAZ signaling pathways. Last, the review explores the potential future directions, stressing that understanding mitochondrial dysfunction is crucial for developing effective therapies to improve mitochondrial function and address related diseases.

## Introduction

Mitochondria are highly dynamic and autonomous organelles within the cell. As the central hubs of energy metabolism, mitochondria are involved not only in adenosine triphosphate (ATP) synthesis but also in the biosynthesis of fatty acids and the generation of reactive oxygen species (ROS). Beyond these metabolic functions, mitochondria are integral to a spectrum of signal transduction pathways, including oxidative stress, cell differentiation, apoptosis, and mitochondrial autophagy [1]. These features and functions render mitochondria a multifunctional central hub, essential for maintaining the normal physiological functions of the cells. The highly variable shapes, numbers, sizes, and locations resulting from mitochondrial fusion, division, mitophagy, and transport are collectively referred to as mitochondrial dynamics [2]. These dynamic processes are essential for maintaining mitochondrial functions and cellular functions [3]. Dysregulation of mitochondrial dynamics has been linked to the development of a variety of diseases, including neurodegenerative disease, metabolic syndrome, cardiovascular disease (CVD), and cancer [4]. Therefore, regulation of mitochondrial dynamics is considered a potential strategy for treating these diseases.

Mitochondrial dynamics vary greatly among different cell types, stages of cell growth, and stressful stimuli [5]. Cells can modify their shape and function in response to different extracellular biochemical and mechanical cues. They adjust to environmental shifts by managing mitochondrial dynamics and function. Historically, research has concentrated on extracellular biochemical signals such as glucose levels, pH values, and calcium concentration [6–9]. However, ongoing studies reveal that the extracellular microenvironment is intricate and variable, encompassing diverse cell types, their secreted factors, and the extracellular matrix (ECM) [7]. It is established that the mechanical characteristics of the matrix, including stiffness and viscoelasticity, can trigger mechanosensitive receptors and sensors on cell membranes [10]. These mechanical characteristics influence cell behavior and fate via intracellular mechanotransduction pathways. Given that diseases such as cardiovascular conditions and cancers often involve ECM fibrosis, targeting matrix stiffness has emerged as a potent therapeutic strategy [11]. The cytoskeleton, responsible for cell shape and movement through its assembly and disassembly, is crucial in the malignant migration of tumor cells. It also plays a crucial role in controlling mitochondrial dynamics and function [12].

In this review, we initially elucidate the critical role of mitochondrial dynamics and function in disease progression, subsequently focusing on how cells perceive extracellular mechanical signals to modulate mitochondrial dynamics and function through mechanotransduction, as well as the involvement of the cytoskeleton in regulating these processes.

## Mitochondrial Dysfunction and Diseases

**Fig. 1.**
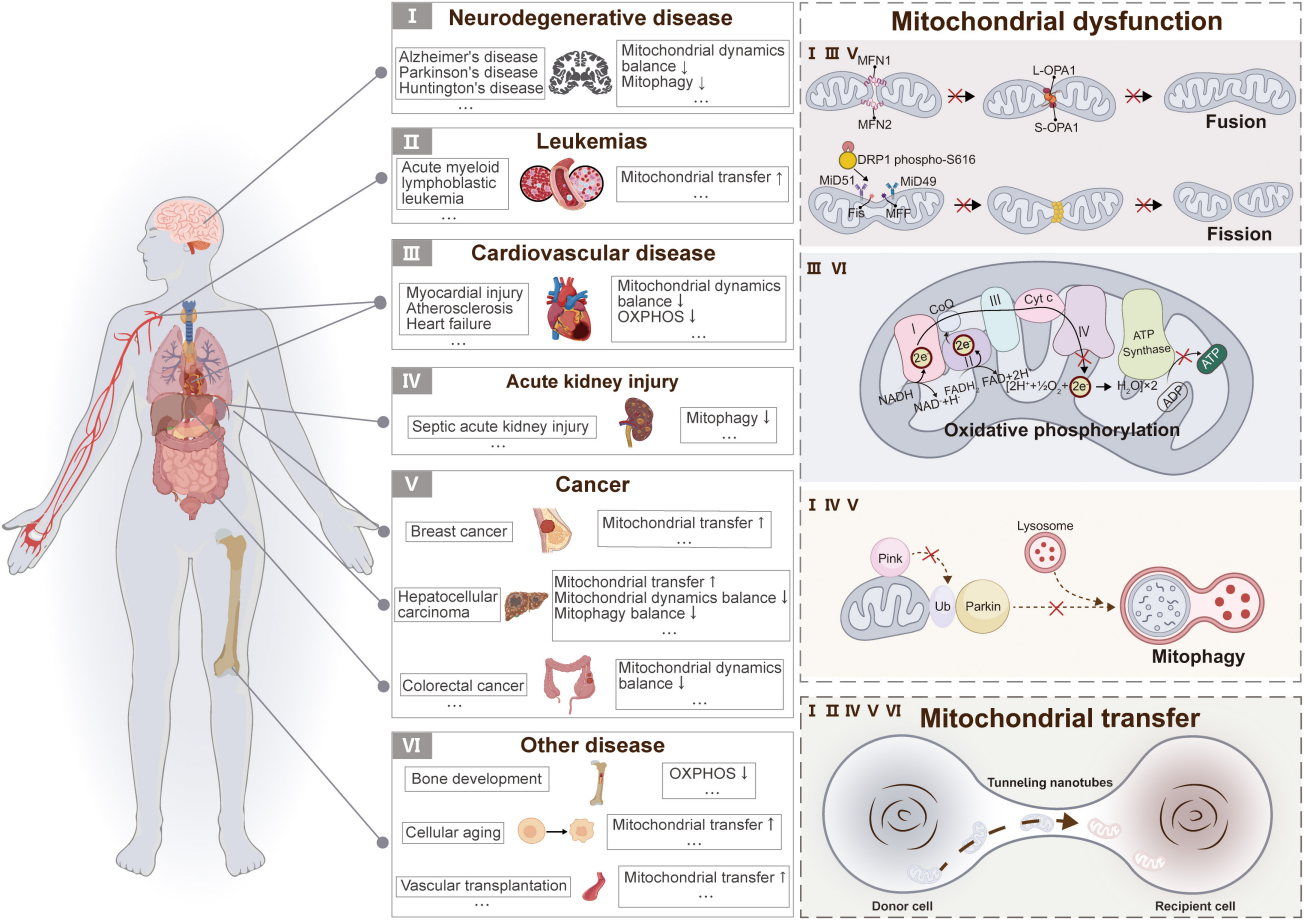
Dysfunctional mitochondria and diseases. Mitochondrial dysfunction includes imbalances in mitochondrial dynamics, impaired mitochondrial OXPHOS, and abnormal mitophagy. In addition, mitochondria can be transferred to other cells via TNTs. Mitochondrial dysfunction has been associated with a variety of diseases characterized by different mitochondrial dysfunctions. I: Neurodegenerative diseases, such as Alzheimer’s, Parkinson’s, and Huntington’s, have been associated with an imbalance in mitochondrial dynamics and a decrease in mitophagy. II: Leukemias, such as AML, have been associated with an increase in intercellular mitochondrial transfer. III: CVDs such as myocardial injury, atherosclerosis, and HF are associated with impaired OXPHOS and imbalanced mitochondrial dynamics. IV: AKI has been associated with reduced mitophagy. V: Cancers such as breast cancer, hepatocellular carcinoma, and colorectal cancer display mitochondrial dysfunction. These defects involve pathological alterations in mitochondrial transfer, dynamics, and mitophagy. VI: Other diseases, such as bone development, aging, and vascular transplantation, show impaired OXPHOS and increased mitochondrial transfer.

To maintain the stability and balance of cellular energy metabolism, mitochondria can flexibly respond to functional demands and adjust their internal composition and behavioral patterns through complex mitochondrial dynamics processes [13,14]. This process not only governs the increases or decreases in mitochondrial quantity, shape transformation, and size adjustment but also determines their distribution within the cell [15,16]. Therefore, the dysfunction caused by mitochondrial dynamics will affect the balance of cellular energy metabolism, thereby accelerating the deterioration of CVDs, neurodegenerative disorders (NDs), cancer, etc., which are illustrated in Fig. 1.

ND includes Alzheimer’s disease (AD), Parkinson’s disease (PD), and Huntington’s disease (HD), which is characterized by continuous neuronal degeneration and death, leading to a variety of neurological symptoms. Research has demonstrated that these disorders are accompanied by imbalances in mitochondrial fission and fusion [17–19], along with abnormal mitophagy [20]. In addition, studies of CVD patient samples have shown a significant inverse correlation between oxidative phosphorylation (OXPHOS) levels and the extent of atherosclerotic lesions [21]. Similarly, in heart failure (HF), by improving the mitochondrial OXPHOS process, it effectively alleviated cardiac insufficiency and slowed HF progression [22]. In cancer cells, the state of mitochondria shows a significant imbalance [23,24], which is closely related to the progression of cancer and the formation of drug resistance [25,26]. Although cancer cells prefer to undergo glycolysis as the primary pathway of energy metabolism in an aerobic environment, OXPHOS acts as a complementary pathway to provide the cells with an additional source of ATP in certain cancer cells [27]. In addition, if cancer cells regulate mitochondrial mass through mitophagy, they may develop drug resistance [28–30]. Indeed, OXPHOS is also involved in bone development [31]. Mitophagy is a process that contributes to tubular cell survival and protects and maintains renal function in a mouse model of septic acute kidney injury (AKI) [32]. Similarly, Jionoside A1-mediated increase in mitophagy attenuates ischemic/reperfusion injury in ischemic stroke by increasing mitochondrial content and ATP [33]. These studies suggest that targeting the regulation of mitochondrial dynamics and function could be an effective target for ameliorating and treating these diseases.

## Mitochondrial Transfer

**Fig. 2.**
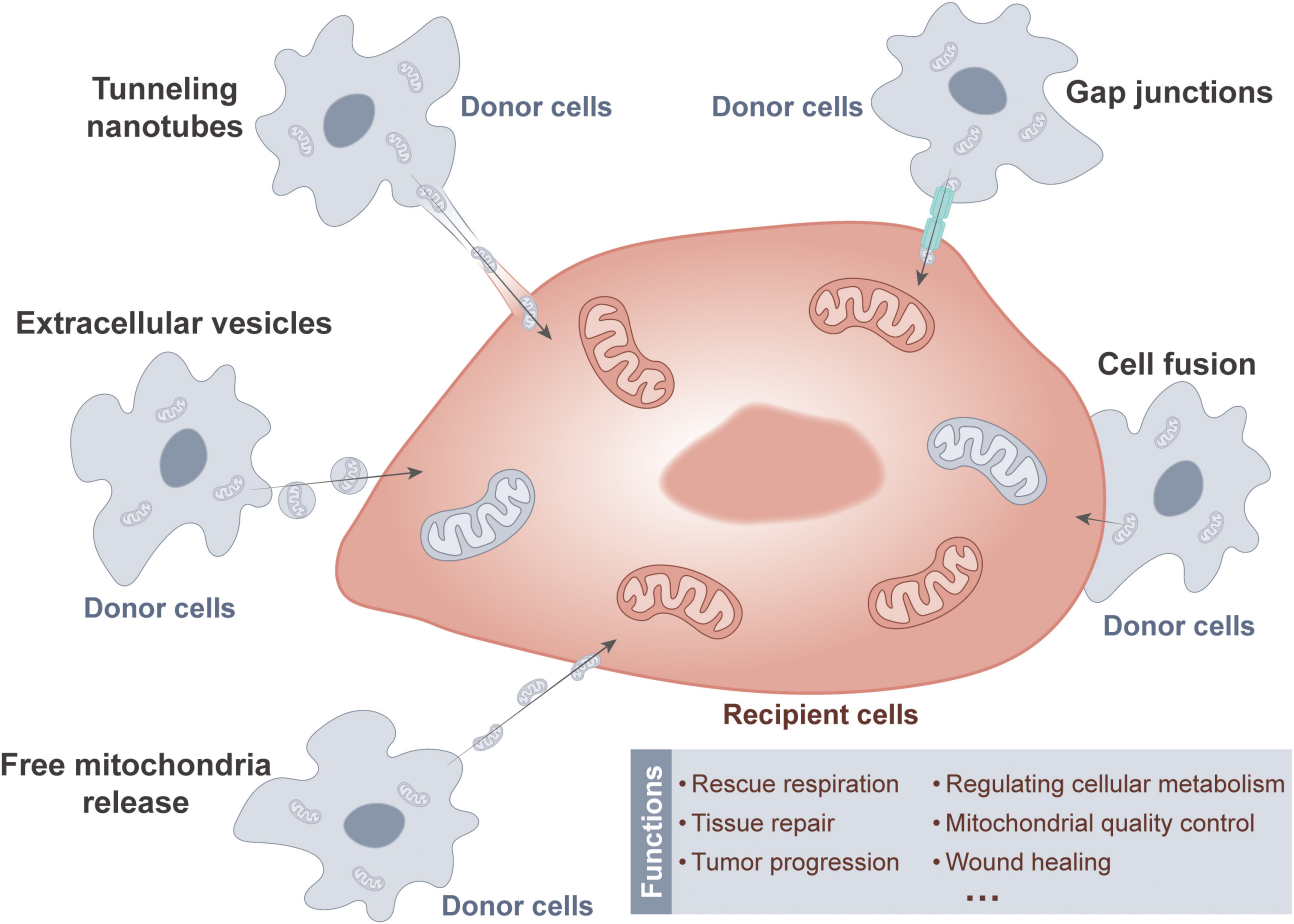
Common modes and routes of mitochondrial transfer. Mitochondrial transfer can be achieved by intercellular contacts such as cell fusion, gap junctions, and TNTs. In addition, mitochondria can be translocated into other cells via extracellular vesicles or free mitochondrial release. The transfer of mitochondria from donor cells to recipient cells is closely related to rescue respiration, tissue repair, and tumor progression.

Delivery of mitochondria from one cell type to another has garnered widespread attention in recent years [34]. The transfer of mitochondria from donor cells to recipient cells is closely related to rescue respiration, tissue repair, and tumor progression [35]. The methods of mitochondrial transfer between cells mainly include tunneling nanotubes (TNTs), gap junctions, cell fusion, extracellular vesicles, and free mitochondria release [36,37]. Common modes of mitochondrial transfer are outlined in Fig. 2. Among them, TNTs are considered the most extensive method of mitochondrial transfer between cells and are also the current focus of research. TNTs are thought to be primarily composed of filamentous actin (F-actin), forming a membrane-like tubular structure with open ends suspended above the matrix. These structures exhibit diameters ranging from 50 to 1,000 nm and lengths ranging from 10 to 200 μm [38]. Previous research has revealed 2 types of formation patterns for TNTs [39]. In one pattern, a single cell or a pair of cells extends processes that then fuse with another cell, forming the nanotube. Another pattern, 2 cells that were initially in contact form a nanotube by moving away from each other in opposite directions. Nevertheless, the precise mechanism underlying the formation of TNTs remains ambiguous. Notably, TNTs can establish connections between distant cells to transport various materials apart from mitochondria, such as lysosomes, calcium ions, and other proteins [40].

Cell-to-cell mitochondrial transfer via TNTs has been extensively reported to play a role in in vivo and in vitro settings, as well as in a variety of disease states, including cancer [41–46]. For instance, when stressed cells in the early stage of apoptosis, such as ultraviolet-treated rat pheochromocytoma cells (PC12), are cocultured with healthy cells, TNTs form between them [47]. These TNTs facilitate the transfer of functional mitochondria from healthy cells to stressed cells, effectively reversing the early apoptotic process in stressed cells. Similarly, in a coculture model of young and aged granulosa cells (GCs) in humans, the transfer of mitochondria from young to aged GCs via TNTs has been confirmed, improving the mitochondrial bioenergetic function of aged GCs [48]. Moreover, in the bone marrow microenvironment, acute myeloid leukemia (AML) cells receive mitochondrial transfer from mesenchymal stem cells (MSCs) via TNTs, thereby enhancing resistance to OXPHOS inhibitors [49].

Donor cells transfer their healthy mitochondria to stimulated or damaged recipient cells through TNTs. This process helps to restore the bioenergetic characteristics of the recipient cells, enhance cell viability, reduce inflammatory processes, and promote the normalization of cell function [50]. Consistent with this concept, cancer cells use TNTs to acquire immune cell mitochondria, enhance their own metabolism, weaken immunity, and accelerate proliferation and immune evasion [51]. A recent publication also reported that cancer-associated fibroblasts (CAFs) exchange goods with breast cancer cells through TNTs, wherein the mitochondria of CAFs serve as critical cargo, significantly enhancing the 3-dimensional (3D) migratory capacity of cancer cells [52]. Interestingly, a recent study reported that low invasive hepatocellular carcinoma (HCC) acquires mitochondria from highly invasive HCC through TNT mechanism, which significantly enhances the migration and invasion capabilities of low invasive HCC [53]. The acquisition of mitochondria by TNTs is beneficial for cancer cell invasion. This cellular process may also be a novel target for cancer therapy.

In addition to the fact that the transfer of mitochondria through TNTs has been intensively studied in the field of cancer, this phenomenon has received similar reports and attention in other fields of biology and medicine. For instance, a study has reported that in the context of vascular transplantation, MSCs can transport mitochondria to endothelial cells (ECs) via TNTs [54]. This process is crucial for the successful implantation and functioning of ECs in vivo, and thus offers significant clinical potential. Another study suggests that promoting the formation of TNTs between senescent bone marrow mesenchymal stromal cells (BMSCs) can accelerate the transfer of mitochondria between these cells, thereby restoring mitochondrial function and ultimately significantly enhancing the viability of senescent BMSCs [55]. According to these studies, we deeply realize that mitochondrial transfer through TNTs plays a pivotal role in biology and medicine, and its far-reaching impact and great potential indicate that this field is worthy of further exploration and research.

## Cues from Biochemical and Mechanical Sources Reorganize and Regulate Mitochondrial Networks and Functions

**Fig. 3.**
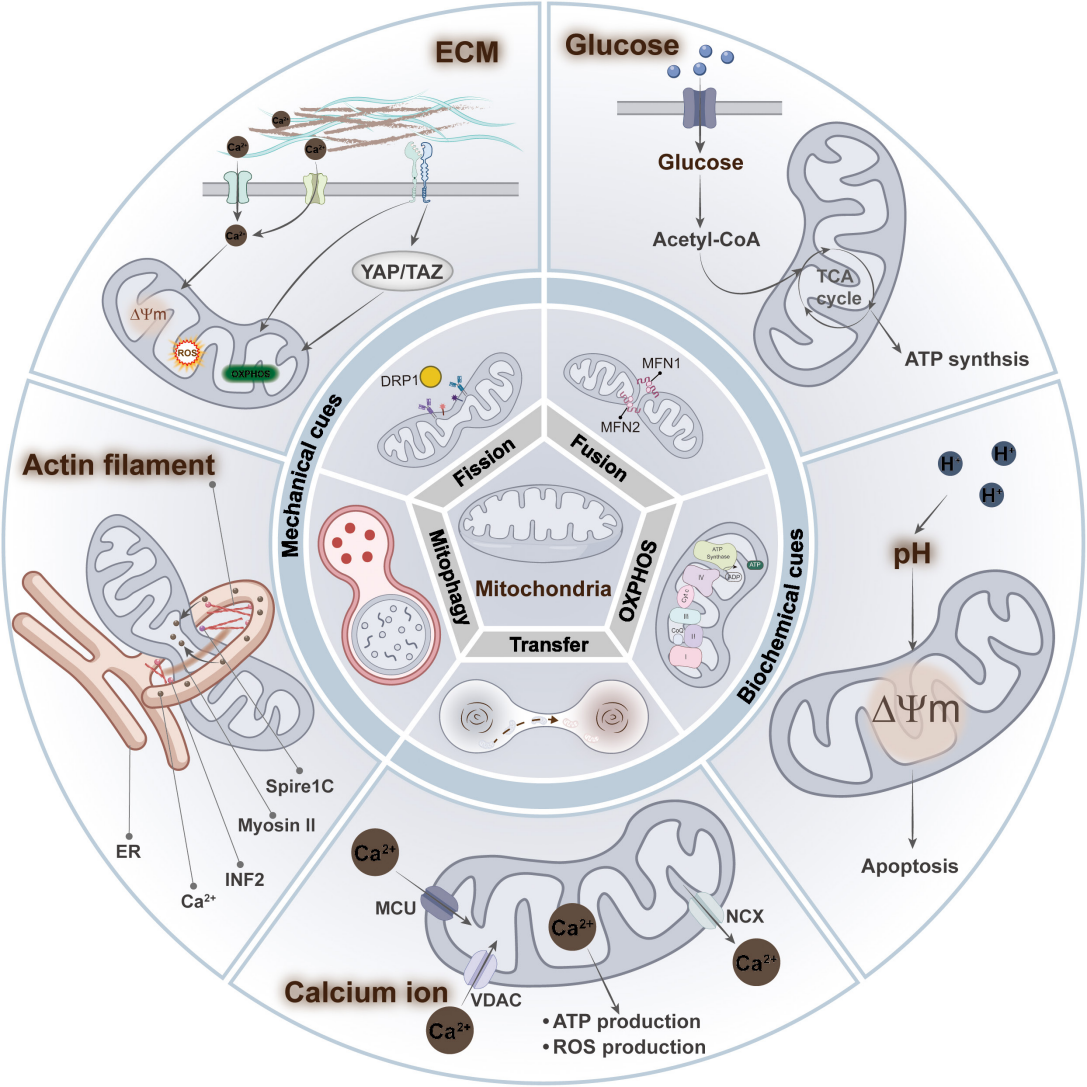
Biochemical and mechanical cues reorganize and regulate mitochondrial networks and functions. Biochemical cues include extracellular glucose concentration, pH, and Ca^2+^ concentration. Glucose concentration can regulate mitochondrial OXPHOS and ATP production by affecting the tricarboxylic acid (TCA) cycle. Changes in pH can induce alterations in mitochondrial membrane potential, triggering apoptosis via the mitochondrial pathway. On the one hand, Ca^2+^ can promote ATP production. On the other hand, Ca^2+^ overloaded in mitochondria may lead to elevated ROS levels. Mechanical cues include the physical properties of the ECM and intracellular cytoskeleton dynamics. ECM stiffness cause changes in mitochondrial dynamics and function by transmitting signals into the cell through mechanosensitive receptors on the cell membrane. The actomyosin network, in turn, is involved in regulating mitochondrial division processes, which indirectly affect mitochondrial function.

The biochemical and mechanical characteristics of the cellular microenvironment are altered by various diseases, and cells sense these mechanical cues to regulate their morphology, function, and metabolism [7]. Therapeutic strategies targeting the extracellular microenvironment hold promise for addressing the progression of various diseases, particularly in cancer [56]. There is growing evidence that mitochondrial dysfunction results from abnormal changes in both the biochemical composition and physical properties of the microenvironment. We will then discuss how these cues, including biochemical cues such as glucose concentration [9], potential of hydrogen (pH) [57], and Ca^2+^ [58] and mechanical cues such as stiffness [59], influence cellular activity, especially their role in controlling mitochondrial dynamics and functions (Fig. 3).

### Biochemical cues

#### High glucose

High glucose, as a biochemical stressor, induces a rapid shift in mitochondrial morphology from filamentous networks to fragmented structures in human mammary epithelial cells. This process result in increased intracellular ROS levels and increased levels of Ca^2+^ in mitochondria, ultimately triggering oxidative stress [9]. Mechanistically, this may be due to the high glucose-induced increase in intracellular ROS levels, which inhibits adenosine monophosphate (AMP)-activated protein kinase (AMPK) activity and indirectly promotes Dynamin-related protein 1 (DRP1) activation, leading to mitochondrial division [60]. Similarly, high glucose-induced impairment of mitochondrial integrity and reduced mitochondrial respiration, as evidenced by short and small mitochondria, cytoplasmic cytochrome c levels, and reduced mitochondrial ATP levels, were observed in primary retinal cells [61].

#### pH

The changes in extracellular pH significantly impact the activity of various ion channels and transporter proteins on the cell membrane, which in turn affects cell function [62]. However, some studies have shown that increasing cellular pH can induce mitochondrial dysfunction-dependent cancer cell death by triggering a series of molecular events. For example, bicarbonate increased intracellular cytoplasmic and mitochondrial pH in liver cancer cells (SK-HEP-1) and induced mitochondrial persistent permeability (MPT) and mitochondrial damage, which resulted in a disruption of OXPHOS. It leads to a significant increase in AMP, which activates classical AMPK-mediated autophagy, leading to cell death [57]. Exposure to high pH buffers raises the pH of the mitochondrial matrix, inhibiting mitochondrial respiration and increasing ROS production. On the one hand, this may have led to spontaneous transient depolarization of mitochondria to reduce ROS production by suppressing continued elevated matrix pH and protecting cells from damage [6].

#### Calcium levels

Notably, a growing number of studies have demonstrated a reciprocal regulatory relationship between Ca^2+^ and mitochondria. On the one hand, mitochondria are involved in the regulation of intracellular Ca^2+^ and Ca^2+^ signaling through their capacity to uptake and store Ca^2+^ [58]. Conversely, Ca^2+^ has been identified as the signature stimulatory signal for the activation of several mitochondrial enzymes that enhance respiratory chain activity [63]. In the presence of Piezo1, the intracellular concentration of Ca^2+^ is increased, which leads to an increase in mitochondrial oxygen consumption and ATP levels [64].

However, when there is an excess of intra-mitochondrial Ca^2+^, this can result in the opening of the mitochondrial permeability transition pore (mPTP), which leads to mitochondrial fragmentation and increased mitochondrial autophagy [65,66]. Additionally, the uptake of calcium ions by mitochondria can result in the production of ROS [67]. Interestingly, the high uptake of mitochondrial Ca^2+^ in the above study did not result in elevated ROS. Most studies have suggested that Ca^2+^-induced ROS elevation is associated with its impact on OXPHOS. These suggest a complex crosstalk between Ca^2+^ and the ROS pathway, the exact regulatory mechanism of which remains unclear.

### Mechanical cues

#### Mechanical microenvironment of ECM

**Fig. 4.**
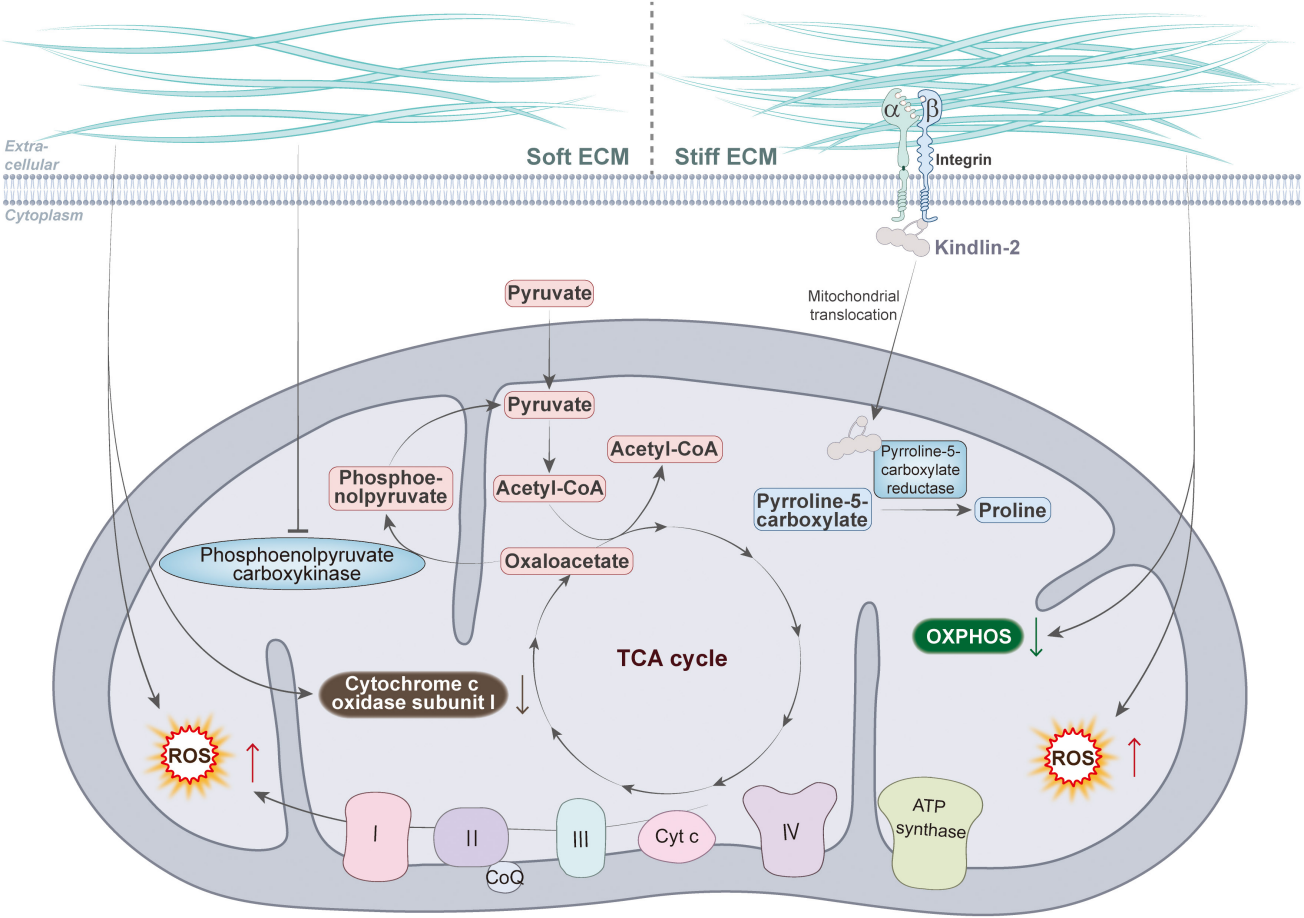
The relationship between ECM stiffness and mitochondrial metabolism. Softer matrix stiffness induced an increase in mitochondrial ROS levels and decrease in ATP levels in breast cancer cells. Mitochondrial PCK2 expression increased with increasing matrix stiffness, which activated glycolysis to promote osteogenesis in MSCs. In lung adenocarcinoma, stiffening of the ECM promotes translocation of kindlin-2 to mitochondria and interaction with the enzyme PYCR1, which induces an increase in proline synthesis and cellular proliferation in tumor cells. In human mammary epithelial cells, the morphology of mitochondria gradually changes as the matrix stiffness increases, which leads to increased intracellular ROS levels and decreased ATP levels. During the spreading of MSCs on the stiff matrix, there is an increase in mitochondrial division and decrease in ATP levels, which increases osteogenic differentiation of MSCs. In HCC cells, mitochondria were fragmented and granular in the softer environment, with down-regulation of mitochondria-encoded cytochrome c oxidase I, which inhibited cell proliferation.

The mechanical forces provided by the ECM have been largely overlooked and have recently been shown in many studies to play a key role in disease progression [68]. ECM is not only a matrix for cell attachment and migration but also a dynamic signaling center that regulates various physiological functions of cells by providing mechanical, chemical, and other stimuli. Considerable articles have been published showing the association between extracellular mechanical cues, particularly ECM stiffness, and mitochondrial dynamics and function, especially mitochondrial metabolism (Fig. 4). For example, in human mammary epithelial cells, the morphology of mitochondria gradually changes from a filamentous network to a ring-like fragmented one as the matrix stiffness increases, which leads to increased intracellular ROS levels and decreased ATP levels [9]. During the spreading of MSCs on the stiff matrix, there is an increase in mitochondrial division and decrease in ATP levels, which increases YAP/TAZ-mediated osteogenic differentiation of MSCs [69]. Similarly, mitochondrial phosphoenolpyruvate carboxykinase (PCK2) expression increased with increasing matrix stiffness, which activated glycolysis to promote osteogenesis in MSCs [70]. These findings imply a multifaceted regulatory relationship between ECM stiffness and mitochondrial dynamics and function during the development of bone tissue.

The development of cancer is usually accompanied by stiffening of the tissues. In lung adenocarcinoma, stiffening of the ECM promotes translocation of kindlin-2 to mitochondria and interaction with the enzyme pyrrolidine-5-carboxylate reductase 1 (PYCR1), which induces an increase in proline synthesis and cellular proliferation in tumor cells. This can be reversed by knockdown of kindlin-2, which serves as an effective strategy to inhibit tumor growth [71]. Similarly, in HCC cells, mitochondria were fragmented and granular in the softer 3D collagen environment, with reduced depolarization of the mitochondrial membrane potential and down-regulation of mitochondria-encoded cytochrome c oxidase I, which inhibited cell proliferation [72]. Softer matrix stiffness induced an increase in mitochondrial division and intracellular ROS levels in breast cancer cells [73]. These studies reveal new targets for inhibiting tumor growth and metastasis by regulating mitochondrial dynamics and function in response to ECM stiffness.

#### Cell mechanics

Mechanical forces acting on cells affect cell morphology, gene expression, and physiological function by regulating cytoskeletal dynamics [74,75]. In this process, the cytoskeleton plays a very important role, which forms a stable molecular network structure within the cell to generate mechanical forces to maintain cell morphology. In particular, actin filaments are the main generators of intracellular mechanical forces and are important for maintaining cell morphology and function [76]. The dynamics of actin filaments have been shown to be involved in a variety of life activities, especially tumor cell proliferation [77], motility [78], and mitochondrial division [79]. An increasing number of studies have demonstrated that the dynamics of the cytoskeleton play a crucial role in regulating mitochondrial morphology. It has been shown that the actin cytoskeleton can be involved in mitochondrial division in several ways. Firstly, by directly affecting mitochondrial division: An advanced technique called platinum replica electron microscopy (PREM) was used to visualize the structure of the cytoskeletal network and mitochondria. The researchers found that actin filaments cross the constrictions of mitochondria that are about to divide [80]. This particular network structure is associated with mitochondrial division, which may exert a tangential force on the mitochondria to promote their division. Secondly, by indirectly affecting mitochondrial division: Overexpression of the splice isoform of spire type actin nucleation factor 1 (Spire1C), which is specifically localized to mitochondria, promotes actin assembly around mitochondria by directly binding to and stimulating inverted formin 2 [INF2; an actin nucleation factor located on the endoplasmic reticulum (ER)] [81]. This leads to an increase in mitochondria–ER contact sites and increased mitochondrial division [82].

Actin works in conjunction with myosin to fulfill its function. Myosin binding and gliding can regulate the dynamics of actin network and influence the remodeling of the cytoskeleton [83]. Intriguingly, a number of studies have shown that myosin can be indirectly involved in mitochondrial division by interacting with actin [12]. For example, the mitochondria-localized myosin 19 tethers mitochondria to ER-associated actin to enhance ER contact with mitochondria and promote mitochondrial fission [84]. In addition, the contractile activity of the non-muscle myosin IIA (NMIIA) leads to stochastic inhomogeneous deformations of the cytoskeletal network. This highly dynamic mechanically active actin filament exerts a localized squeeze on mitochondria, which leads to localized invagination of the mitochondrial surface. This may be a critical point for initiating mitochondrial fission [80]. In general, actin and myosin play a key role in mitochondrial division and dynamic balance by remodeling the cytoskeletal network, recruiting division proteins, transporting and localizing them, and regulating mitochondrial morphology. This regulation of mitochondrial dynamics is important for cellular energy metabolism, signaling, and other processes.

### Mechanotransduction in mitochondrial dynamics and function

Cells sense extracellular mechanical cues through different mechanisms, causing a series of biochemical reactions downstream within cells that ultimately affect various cellular behaviors; these processes are called mechanotransduction. Previously, we introduced that mechanical cues affect intracellular mitochondrial dynamics and function. In the subsequent sections, we will delineate the process of mechanotransduction that ensues when cells are subjected to mechanical stimuli, emphasizing the consequential effects on mitochondrial function and dynamics.

#### Mechanosensors

Cells respond to mechanical signals through transmembrane proteins localized to the cell membrane or proteins that sense changes in membrane tension caused by these signals. These proteins then activate downstream effector proteins, which ultimately cause spatial and temporal changes in gene expression, thereby regulating cellular function [85,86]. For example, cells can sense ECM stiffness through integrins, which are activated to transmit signals into the cell, causing changes in the dynamics of the cytoskeleton [87]. The cytoskeletal remodeling events in turn affect various cellular functions [76]. In addition, there are a number of mechanosensitive ion channels on the cell membrane in addition to adhesion receptors, such as Piezo1, a member of the Piezo family of mechanically activated cation channels. Piezo1 senses changes in membrane plasmodesmata caused by mechanical signals and transmits them to the cell, realized as changes in Ca^2+^ concentration [88]. This signaling process is involved in the regulation of various cellular physiological activities, such as cell migration, proliferation, and differentiation [89]. Collectively, these mechanosensitive molecules ultimately regulate cellular physiological activities such as gene expression, cell proliferation, differentiation, and apoptosis by participating in signal transduction pathways.

In addition to its role as an energy factory within the cell, mitochondria also play key roles in a variety of signaling pathways [4]. For example, mitochondria are capable of taking up and releasing Ca^2+^, which act as important second messengers in a variety of signaling cascades [58]. In addition, ROS produced by mitochondrial metabolism can act as signaling molecules to regulate cellular processes [90]. Mitochondrial metabolites such as nicotinamide adenine dinucleotide (NAD^+^) and adenosine diphosphate (ADP)/ATP can sense cellular energy status and transmit signals [91]. Eventually, signaling cascades such as the mitochondrial unfolded protein response are activated when mitochondrial dysfunction occurs [92]. Overall, mitochondria play an important role in the cellular signaling network and are involved in the regulation of a variety of key physiological processes.

**Fig. 5.**
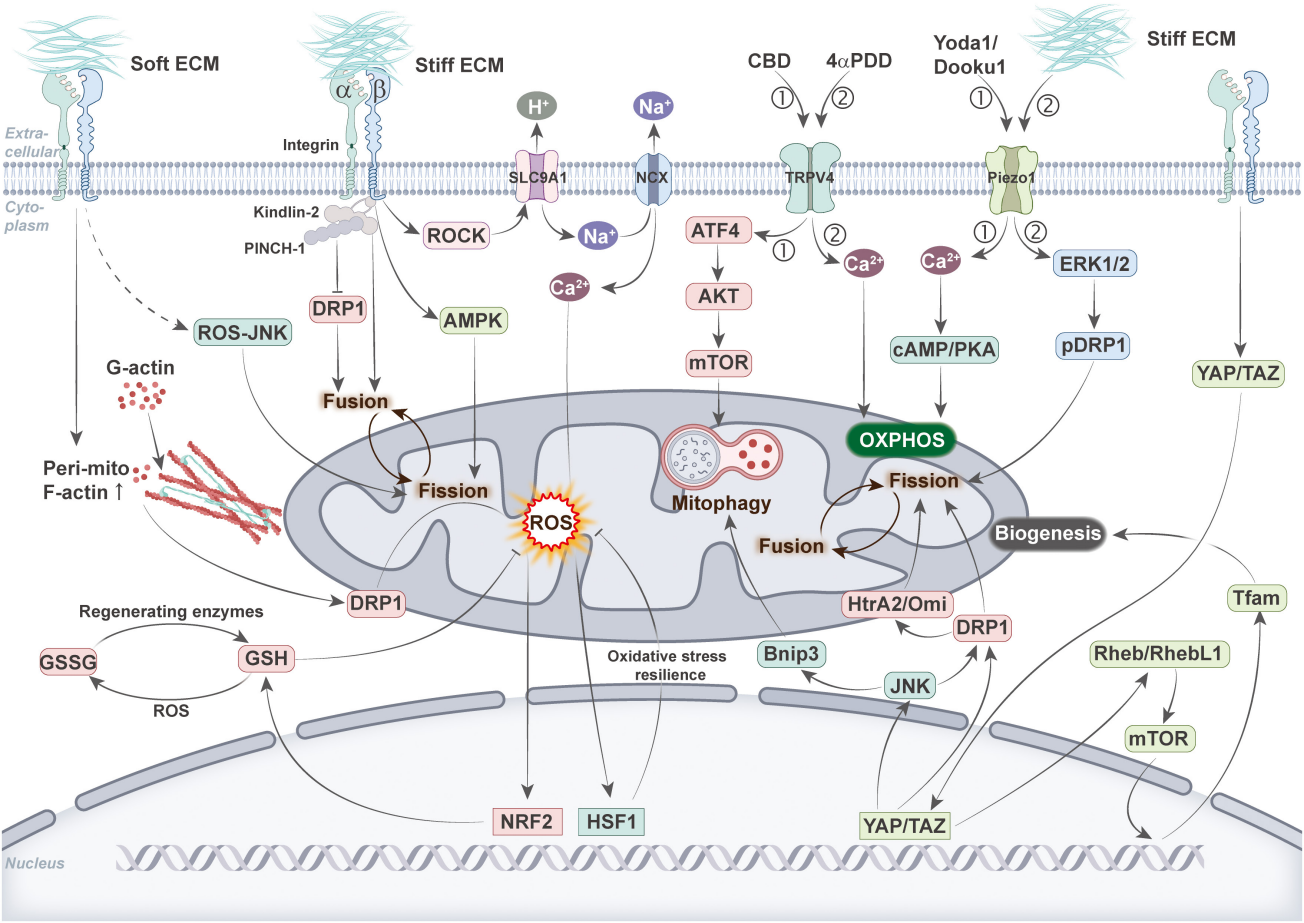
Integration of mechanotransduction pathways and mitochondrial dynamics and function. Mechanotransduction pathways can be divided into 3 categories: the integrin pathway, the Piezo1/TRPV4 pathway, and the YAP/TAZ pathway. Cells sense alterations in ECM stiffness through integrin receptors. The soft matrix promotes mitochondrial division by polymerization of actin around mitochondria. This activates the ROS–NRF2 pathway and promotes glutathione production to counteract oxidative stress. Soft matrix also promotes mitochondrial division by activating the ROS–JNK pathway. On the other hand, hard matrix regulates mitochondrial fusion and division by interacting with kindlin-2 and PINCH-1 of integrins, or via AMPK. The hard matrix also regulates the activity of SLC9A1 and NCX by the integrin–ROCK pathway, which overloads mitochondrial calcium ions and activates the ROS–HSF1 pathway. HSF1 activates the expression of genes related to antioxidative stress, which helps the cells to fight against oxidative stress. Piezo1 and TRPV4 are classes of mechanosensitive ion channels. The activation of TRPV4 allows the influx of calcium ions into the cell. This can promote mitochondrial OXPHOS and also trigger mitochondrial autophagy through the ATF4/AKT/mTOR pathway. Similarly, activation of Piezo1 also allows calcium ion influx, which, on one hand, promotes OXPHOS via the cAMP/PKA pathway and, on the other hand, induces DRP1-mediated mitochondrial division via the ERK1/2 pathway. YAP/TAZ is a class of mechanosensitive transcription factors. Activated YAP enters the nucleus and affects mitochondrial division by regulating DRP1 activity, which may also regulate mitochondrial autophagy via JNK/Bnip3. In addition, activated TAZ enters the nucleus and regulates mitochondrial biogenesis through the Rheb/RhebL1–mTOR–Tfam pathway.

Therefore, it is reasonable to question whether mitochondria are involved in mechanotransduction processes or extracellular mechanical forces modulate mitochondrial function to influence cellular behavior. Certainly, there have been many studies demonstrating that cellular mechanical forces can regulate mitochondrial function through different mechanisms (Fig. 5 and Table).

**Table.** Summary of the mechanotransduction pathways related to mitochondrial morphology, function, and their impact on cell fate

Influencing factors	The effect on mitochondria	Mechanisms involved	Results	References
Stiff ECM	Fusion	Integrin–kindlin-2/PINCH-1	Cell spreading area	[94]
Stiff ECM	Fission, ROS↑	Integrin–Ca^2+^–HSF1	Oxidative stress resilience	[9]
Stiff ECM	Fission, OXPHOS	Integrin–AMPK	Osteogenic differentiation	[69]
Soft ECM	Fission	ROS–JNK	Apoptosis	[101]
Soft ECM	Fission, mitophagy	IP3R–Ca^2+^–pDRP1	Cell survival	[66]
Soft ECM	Fission, ROS↑	Integrin–ROS–NRF2– glutathione	Oxidative stress resilience	[73]
Yoda1/Dooku1	OXPHOS	Piezo1–Ca^2+^–cAMP/PKA	ATP↑	[109]
Stiff ECM	Fission	Piezo1–ERK1/2–pDRP1	Apoptosis	[110]
4αPDD	OXPHOS	TRPV4–Ca^2+^	Osteoblast differentiation	[112]
CBD	Mitophagy	TRPV4–ATF4–AKT–mTOR	Cell death	[113]
Yoda1	Depolarization	Piezo1–TRPV4–Ca^2+^	Cell death	[115]
YAP deficiency	Mitophagy	YAP–JNK–Bnip3	Cell migration	[119]
TAZ knockout	Biogenesis	TAZ–Rheb/RhebL1–mTOR–Tfam	Impaired exercise ability in mice	[120]
YAP deficiency	Fission	YAP–JNK/DRP1–HtrA2/Omi	Apoptosis	[121]
Stiff ECM	Fission, biogenesis	YAP–DRP1	Osteogenic differentiation	[126]

##### Integrins

Cell–ECM interactions are primarily facilitated by integrins and transmembrane receptors for ECM proteins. The binding of integrins to ECM proteins recruits a multitude of adhesion proteins, such as talin, kindlin-2, paxillin, integrin-linked kinase (ILK), and PINCH-1 [93], to cell–ECM adhesion sites where they transmit signals to downstream effectors and influence cellular behavior. A study has demonstrated that alterations in mitochondrial dynamics induced by ECM rigidity in human MSCs are mediated through 2 distinct integrin-dependent signaling pathways: specifically, the up-regulation of mitochondrial fusion via kindlin-2 and the inhibition of DRP1 expression and subsequent mitochondrial fission through PINCH-1 [94]. High matrix stiffness is thought to activate integrins, which will activate a series of intracellular signaling pathways to regulate physiological processes such as cell proliferation, migration, and survival [87]. Interestingly, in human mammary epithelial cells, high matrix stiffness activates the integrin–rho-associated protein kinase (ROCK) signaling pathway, which regulates the activity of the H^+^ transporter protein–solute carrier family 9A1 (SLC9A1) and Na^+^/Ca^2+^ exchangers (NCX) at the cell membrane, resulting in mitochondrial Ca^2+^ loading and ROS production [95,96]. Meanwhile, activation of heat shock factor 1 (HSF1) and its target gene expression in a heat stress-independent manner conferred cellular resistance to oxidative stress by limiting mitochondrial respiration and causing mitochondrial reprogramming [97]. The dynamics of cell spreading is the first step in the interaction between the cell and the ECM, which is based on the interaction of integrins with the ECM [98]. Research has shown that MSCs have different mitochondrial morphology and different ATP levels during cell spreading on different matrix stiffness. On stiffer substrates, the cells spread over a larger area, the intracellular mitochondria were in a divided state, and the total ATP level was higher due to the activation of AMPK by the stiff substrate, which in turn regulates mitochondrial function [69].

Tumor progression is accompanied by a continuous hardening of the ECM, while cancer cells usually have more mitochondria to meet the demands of their rapid proliferation and energy metabolism [11,99,100]. However, when tumors metastasize to other tissues, they encounter a softer ECM microenvironment again. A study showed that soft substrates activated DRP1-mediated mitochondrial fission with polymerization of actin around mitochondria in breast cancer cells, resulting in elevated levels of mitochondrial ROS and inducing intracellular oxidative stress. In response to the stress, the cells activated nuclear factor erythroid 2-related factor 2 (NRF2)-mediated expression of genes related to the antioxidant stress response, increasing cystine uptake and glutathione metabolism levels, which, in turn, conferred resistance to ROS-dependent drugs, thereby promoting cell survival [73]. Similarly, soft matrix activated the ROS–JNK (c-Jun N-terminal kinase) signaling pathway in breast cancer cells, which induced apoptosis through the mitochondrial pathway. Interestingly, soft matrix simultaneously induced intracellular protective autophagy, which inhibited apoptosis to promote cell survival [101]. However, this article does not mention integrins, and their upstream mechanisms remain as in need of further study. Significantly, the same conditions, such as a softer ECM, which promoted mitochondrial division in breast cancer cells, had the opposite result in normal cells. This may be influenced by the distinctive metabolic characteristics of cancer cells and the tumor microenvironment. Further investigation is required to validate this finding in other cancer cells.

Transverse aortic constriction (TAC) is one of the most commonly used disease models of chronic ventricular hypertrophy and can be used to mimic hypertrophic cardiomyopathy caused by hypertension or increased intraventricular pressure [102]. Melusin is a chaperone protein that is selectively expressed in the heart and binds to the cytoplasmic region of β1 integrins to transduce mechanical signals [103]. It was found that in a mouse model of TAC, melusin enters mitochondria to bind and inhibit mitochondrial trifunctional protein (MTP), inhibit fatty acid oxidation (FAO), and inhibit ROS production, which protects mice from TAC-induced pressure overload [104]. Mechanistically, this may be related to the mechanotransduction induced by changes in blood flow shear caused by TAC.

##### Ion channels: Piezo1 and TRPV4

Piezo1 and transient receptor potential cation channel subfamily V member 4 (TRPV4) have been demonstrated to promote extracellular Ca^2+^ influx and activate intracellular Ca^2+^-mediated signaling pathways, which in turn affect cellular metabolism and function [105,106]. Under pathological conditions such as hypertension and vascular occlusion, ECs respond to changes in blood flow shear via Piezo1/TRPV4, which triggers an increase in intracellular Ca^2+^ concentration, which in turn regulates processes such as angiogenesis and vasodilation. In addition, Piezo1/TRPV4 has been associated with malignant angiogenesis in tumors [107,108]. Due to the significant interconnection between Ca^2+^ and mitochondria, we aimed to elucidate the relationship between these ion channels and mitochondrial dynamics and function.

The specific Piezo1 channel activator Yoda1 or its analog Dooku1 has been observed to increase the concentration of Ca^2+^ in human umbilical vein ECs (HUVECs) and in mitochondria [64]. This ultimately enhances mitochondrial respiration and glycolysis, stimulating ATP production. Similarly, another study has demonstrated that the activation of Piezo1 results in the uptake of mitochondrial calcium and the subsequent induction of OXPHOS. A more comprehensive investigation revealed that Piezo1 enhances mitochondrial functionality by elevating cAMP signaling [109]. A study has demonstrated that the presence of a stiff matrix stimulates the activation of the Piezo1 channel, which in turn facilitates the influx of calcium ions. This initiates a cascade of events involving the activation of extracellular signal-regulated kinases 1 and 2 (ERK1/2) and the phosphorylation of DRP1, ultimately leading to the fragmentation of mitochondria and the subsequent induction of apoptosis [110].

Similar to Piezo1, TRPV4 has been the subject of extensive study as a mechanosensitive ion channel, particularly in the context of osteoblast research [111]. In osteoblasts, the activation of TRPV4 has been observed to increase intracellular as well as mitochondrial Ca^2+^ levels, and promote basal and maximal mitochondrial respiration and ATP production [112]. This has been demonstrated to facilitate osteoblast differentiation. Moreover, TRPV4 activation has been demonstrated to regulate mitochondrial morphology, although the precise mechanisms involved remain uncertain. Another study indicated the non-psychoactive phytocannabinoid cannabidiol (CBD), a drug used to treat gliomas, possesses a mechanism of action that induces mitochondrial autophagy through the activation of TRPV4, which ultimately results in cell death [113]. Mechanistically, the ATF4 (activating transcription factor 4)–DDIT3 (DNA damage inducible transcript 3)–TRIB3 (tribbles pseudokinase 3)–AKT (serine-threonine protein kinase )–mTOR (mammalian target of rapamycin) pathway [114], which is activated by TRPV4, plays a crucial role in CBD-induced mitochondrial autophagy. It is noteworthy that in pancreatic acinar cells, Piezo1 activation resulted in a transient elevation of intracellular calcium levels, whereas Piezo1 activation-induced opening of TRPV4 channels led to a prolonged elevation of intracellular calcium levels, mitochondrial depolarization, intracellular trypsin activation, and cell death [115]. These studies have identified Ca^2+^ signaling pathway in which Piezo1 and TRPV4 act in concert.

##### YAP and TAZ

YAP and TAZ have attracted intense interest due to their remarkable biological properties in cell differentiation, tissue development, and cancer progression [116,117]. Moreover, the activities of YAP and TAZ as mechanosensitive transcription factors are directly regulated by ECM stiffness, cell shape, and cytoskeletal tension. To illustrate, when cells are cultured on a stiffer ECM, YAP and TAZ exhibit increased nuclear localization and transcriptional activity, whereas on a softer ECM they are repressed and relocated to the cytoplasm [118]. In recent years, a number of studies have demonstrated that YAP/TAZ activity is related to mitochondrial function. For example, deletion of YAP results in inhibition of HCC migration. The mechanism is that knockdown of YAP induces phosphorylation of JNK, which binds to the Bnip3 promoter and promotes Bnip3 expression. Higher Bnip3 activity triggers excessive mitochondrial autophagy, leading to mitochondrial dysfunction and ATP shortage [119]. Similarly, specific knockout of TAZ reduced mitochondrial biogenesis, respiratory metabolism, and locomotor activity in mice, which is related to the Ras homolog enriched in brain (Rheb)/Rheb like 1 (RhebL1)–mTOR–mitochondrial transcription factor A (Tfam) pathway [120]. Furthermore, YAP/TAZ plays a role in the regulation of mitochondrial dynamics. To illustrate, the silencing of YAP has been observed to result in JNK phosphorylation, which in turn induces DRP1 activation and translocation to the mitochondrial surface, thereby initiating mitochondrial fission. Excessive mitochondrial fission has been demonstrated to mediate the leakage of high-human temperature requirement A member (HtrA2/Omi) from mitochondria into the cytoplasm [121]. This results in the triggering of apoptosis in human rectal cancer cells via the mitochondrial apoptotic pathway. On the contrary, another study indicated the down-regulation of YAP was observed to inhibit myofibroblast differentiation and result in a reduction of DRP1 levels. This, in turn, led to the elongation of mitochondria, fusion of mitochondrial network, and collapse of the mitochondrial membrane potential [122].

Moreover, studies have demonstrated that YAP/TAZ directly regulates mitochondrial function in response to mechanical stimuli [123]. The transfer of phosphate to ADP, catalyzed by cytoplasmic creatine kinase type B (CKB), enables the regeneration of ATP, thereby maintaining the local ATP gradient [124]. In a stiff environment, pancreatic ductal adenocarcinoma (PDAC) cells not only support ATP production by increasing mitochondrial activity and OXPHOS but also participate in the phosphocreatine-dependent ATP cycling mechanism by expressing CKB via the integrin–YAP pathway [125]. Furthermore, it has been demonstrated that in the absence of a mineralization-inducing medium, MSCs can respond to alterations in ECM stiffness by modulating mitochondrial dynamics and biogenesis for osteogenic differentiation through the regulation of YAP activity. In the stiff substrate, YAP activation inhibits DRP1 activity and enhances Tfam-dependent mitochondrial biosynthesis, resulting in an elongated and fused mitochondrial network [126].

#### Intracellular mechanotransduction

**Fig. 6.**
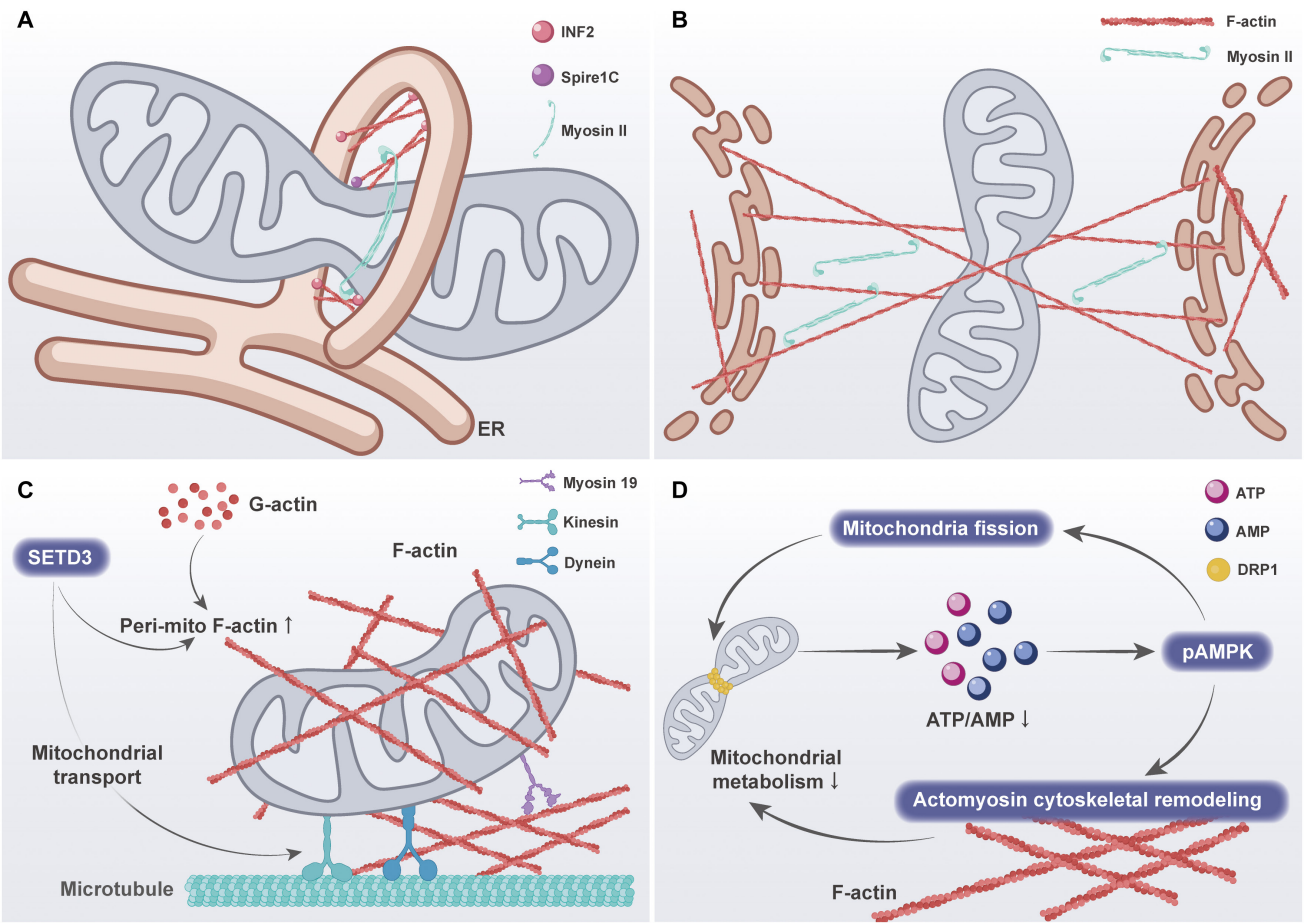
The multiple links between actin and mitochondria. (A) Spire1C specifically localizes to mitochondria and promotes actin assembly around mitochondria by directly binding to and stimulating INF2 on the visceral ER. This leads to an increase in mitochondria–ER contact sites and an increase in mitochondrial fission. In particular, myosin 19 tethers mitochondria to ER-associated actin, enhancing ER–mitochondrial contact and promoting mitochondrial fission. (B) The contractile activity of myosin II leads to random, inhomogeneous deformation of the cytoskeletal network. Mitochondria are locally compressed between taut actin filaments, which result in localized invagination of the mitochondrial surface. This particular network structure may exert tangential forces on mitochondria to promote their division. (C) SETD3, located on the outer mitochondrial membrane, regulates the formation of periplasmic F-actin, which is essential for the maintenance of mitochondrial morphology, movement, and function. (D) Altered mitochondrial metabolic capacity causes changes in the intracellular ATP/AMP ratio, which in turn activates pAMPK. On the one hand, pAMPK regulates cytoskeletal dynamics by direct phosphorylation and inactivation of myosin. On the other hand, pAMPK sustains its continuous activation by regulating mitochondrial division.

In the previous section, we have learned that cytoskeleton remodeling plays a key role in the regulation of mitochondrial dynamics. This suggests that the cytoskeleton may play a major role in the influence of extracellular mechanical signals on mitochondrial dynamics and function. In the following sections, we will describe how the cytoskeleton responds to mechanical signaling to affect mitochondrial dynamics and function (Fig. 6).

SET domain protein 3 (SETD3) is a mechanosensitive enzyme that is localized on the outer mitochondrial membrane and promotes actin polymerization around mitochondria [127]. Loss of SETD3 function resulted in a reduction in peri-mitochondrial F-actin, as well as a reduction in mitochondrial branching length, the number of branches, and mitochondrial movement [128]. These findings provide new insights into the mechanism of peri-mitochondrial F-actin polymerization. Similarly, another study demonstrated that when breast cancer cells are cultured on soft substrates, it is observed that F-actin accumulates around mitochondria in the presence of the actin-related protein 2/3 (Arp2/3) complex and the Spire1C protein, which favors the DRP1-regulated mitochondrial division [83]. Interestingly, further inhibition of ROCK and myosin light chain kinase (MLCK) activity increased the contact of F-actin with mitochondria [73]. This suggests that there may be crosstalk based on different F-actin pools.

Furthermore, as previously stated, AMPK is capable of detecting alterations in the stiffness of the ECM, thereby regulating mitochondrial morphology. There are 2 modes of cell migration across the matrix: elongated mesenchymal and rounded-amoeboid modes of movement. The different migration patterns are related to the levels of intracellular metabolism and actomyosin skeleton activity. One study showed lower levels of adhesion to the substrate and lower levels of ATP within rounded-amoeboid cells, which activated pAMPK [129]. Activation of AMPK results in the direct phosphorylation and inactivation of myosin phosphatase, which in turn leads to increased phosphorylation of myosin light chains and increased overall myosin II activity. These determine the pattern of cell movement and the level of adhesion to the substrate. Concurrently, AMPK preserves the balance in energy levels by inducing mitochondrial fission, which further promotes AMPK signaling and myosin II activation. These studies demonstrate that mitochondrial dynamics can indirectly regulate the cytoskeleton by altering energy levels.

The actin skeleton plays a role in the regulation of mitochondrial dynamics, as well as in the processes of mitochondrial transport and localization. Integrin-based mechanosensing of ECM stiffness has been observed to result in the formation of diffuse actin structures in cells exposed to soft ECM, while cells on hard ECM exhibit prominent actin stress fibers [69]. This phenomenon has been linked to increased mitochondrial motility and reduced perinuclear localization of mitochondria in comparison to those observed in cells on soft ECM [130].

#### Motor-driven mitochondrial transfer along the cytoskeleton

To explore the mechanical signals involved in the transfer of mitochondria between cells via TNTs, the research has concentrated on the biomechanical changes occurring within cells. Therefore, a deeper understanding of the composition, structure, and formation mechanism of TNTs is essential for elucidating the mechanical signal transmission during this process.

**Fig. 7.**
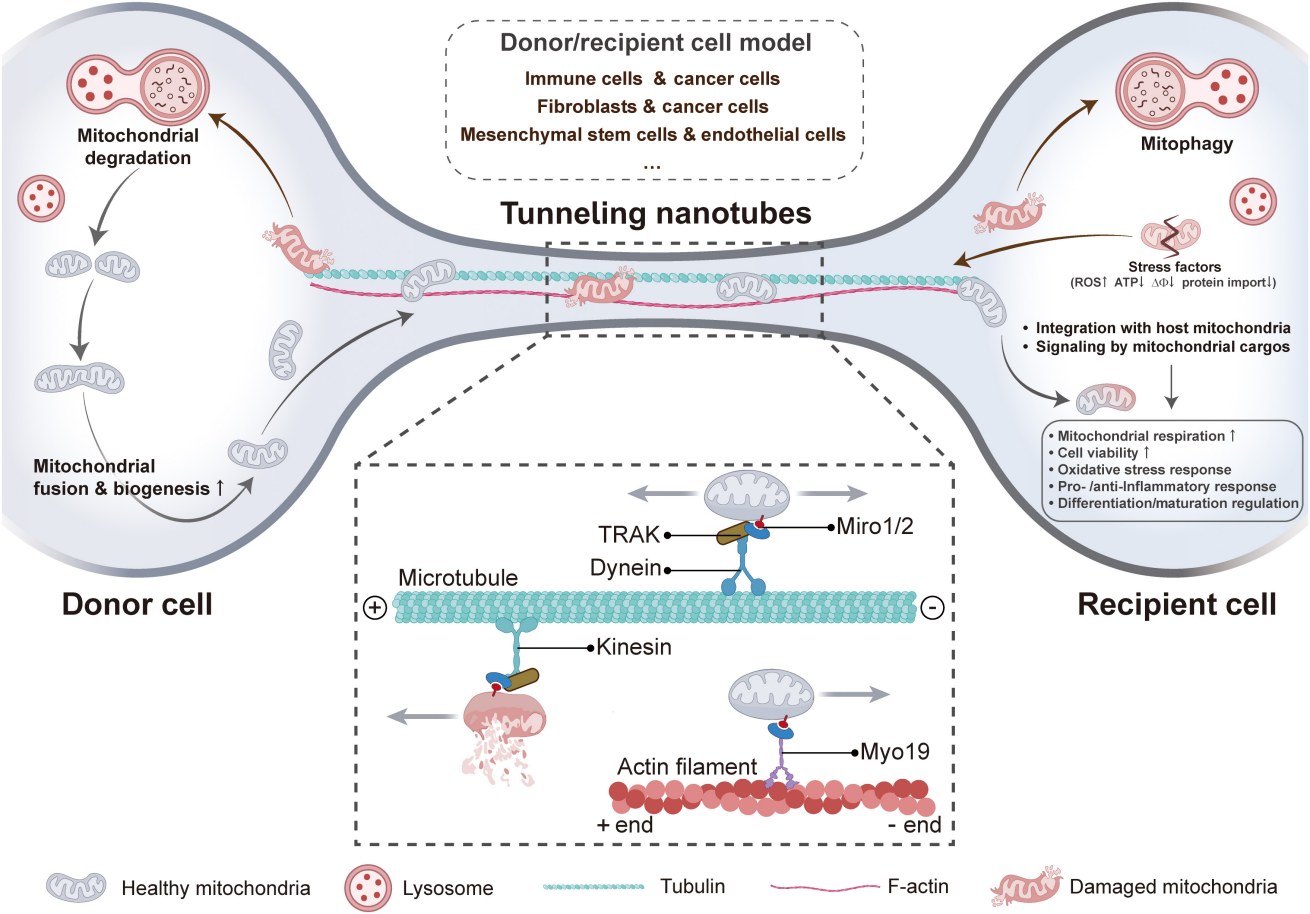
Mechanotransduction in mitochondria transfer. Mitochondria can be transferred between cells via TNTs. This can occur between immune cells and cancer cells, fibroblasts and cancer cells, MSCs and ECs, and other homogeneous and heterogeneous cells. The donor cells transfer their healthy mitochondria via TNTs to the stimulated or damaged recipient cells. This process helps to restore the bioenergetic properties of the recipient cells, enhances cellular viability, reduces inflammatory processes, and promotes normalization of cellular functions. Similarly, damaged mitochondria that are transferred to donor cells are used for other mitochondrial fusion processes and mitochondrial biogenesis through degradation. Mitochondria exhibit 2 modes of movement in TNT: one is microtubule-driven transport and the other is actin-based transport. When mitochondria form a motor articulation complex through Miro1/2, TRAK, and dynein, it can move in both directions along microtubules. When bound to kinesin, it can only move along the plus end of the microtubule. In addition, mitochondria through Miro1/2 can also synergize with Myo19, which allows it to move along the minus end of actin.

Mitochondrial transport in TNTs depends on the action of dynein and kinesin motor complexes that move along the cytoskeleton, such as F-actin and microtubules (Fig. 7). In particular, these motor complexes contain adaptor proteins such as Mitochondrial Rho guanosine triphosphatases (GTPases) 1 and 2 (Miro1 and Miro2), which are tightly anchored to the mitochondrial outer membrane and effectively facilitate unidirectional or bidirectional mitochondrial movement on the cytoskeleton within the TNTs [36,131]. Intriguingly, a recent study showed that mitochondrial precursor proteins accumulated in the cytoplasm did not migrate spontaneously in TNTs, indicating that mitochondrial transport through TNTs depends on an active mechanism rather than a simple passive diffusion [132].

Mitochondria exhibit 2 modes of intracellular movement: one is long-distance transport driven by microtubule mechanisms, and the other is short-distance actin-based transport [133]. Specifically, mitochondrial transport through TNTs is mediated by mitochondrial Miro1, allowing it to move along the cytoskeleton within TNTs. Mechanically, Miro1 acts synergistically with the auxiliary proteins Miro2, transport-associated protein 1 and 2 (TRAK1 and TRAK2), and myosin 19 (Myo19) to drive mitochondrial binding to kinesin family motor protein 5 (KIF5)-driven proteins [134]. When combined, they form a motor adaptor complex that facilitates the transport of mitochondria within the TNTs along the cytoskeleton and regulates their motility.

The mechanism of mitochondrial transport within TNTs is not exclusively reliant on a singular cytoskeletal element [135]. Consequently, in F-actin-only TNTs, mitochondria are predominantly transported along actin filament. In contrast, in TNTs that incorporates both F-actin and microtubules, mitochondrial transport may exhibit greater flexibility, potentially utilizing either F-actin or microtubules—or even both simultaneously. This observation further highlights the intricate complexity and adaptability inherent to mitochondrial transport within TNTs. If mitochondria are transported along F-actin within TNTs, it is critical that Miro proteins recruit and stabilize Myo19 on mitochondria, as Myo19 is a key molecule linking mitochondria to the actin cytoskeleton. Myo19 directly interacts with Miro to participate in mitochondrial transport without the involvement of other complexes. By contrast, Miro cooperates with KIF5 and TRAK to form a complex during mitochondrial transport along TNT microtubules [136]. The complex effectively drives mitochondrial movement along the microtubules of TNTs under the regulation of KIF5. For instance, between cardiomyocytes and cardiac fibroblasts, mitochondria are efficiently transported along microtubules by TNTs using KIF5 [46].

Mitochondrial movement mediated by kinesin and dynein motors on microtubules enables efficient bidirectional movement along microtubules [137]. The KIF family is responsible for the directed transport of mitochondria along the plus end of microtubules, while dynein drives mitochondria along the minus end of microtubules [138]. The adaptor proteins Milton and Miro interact to become a key bridge connecting mitochondria to motor proteins, regulating the bidirectional movement of mitochondria on microtubules [139]. Through the tight association of Miro with Milton, motor proteins, particularly kinesin-1, are efficiently recruited to mitochondria, leading to efficient mitochondrial transport along microtubules. Furthermore, Miro1 is required by TRAK2 for minus-end directed mitochondrial transport in fibroblasts. However, in the presence of overexpression of KIF5, TRAK2 regulates plus-end directed transport of mitochondria even in the absence of Miro1. Remarkably, TRAK1 and TRAK2 were still able to localize to mitochondria even in the absence of Miro1 and Miro2 [136]. This suggests that their binding to mitochondria may not be entirely dependent on Miro proteins or that other unknown interaction mechanisms exist.

It is important to note that we have identified an unusual member of the myosin family, human Myo19. This protein is unique in that it is specifically anchored to mitochondria through its tail domain, but is not directly inserted into the mitochondrial outer membrane [140]. In contrast, human Myo19 was shown to bind tightly to the Miro GTPase, and this association constitutes an important bridge between the myosin motor and mitochondria, thereby enabling their coordination and connection [136]. Consistent with these findings, a study reported that Myo19 dimers exhibit efficient transport ability on actin tracks in the cell. These findings not only reveal the intracellular motility properties of Myo19 molecules but also provide strong evidence that Myo19 molecules are able to transport mitochondria directly on actin tracks and are essential for maintaining cellular function and homeostasis. In addition, the importance of actin dynamics in mitochondrial transport has been illustrated in numerous review articles [135,139]. Although the process of mitochondrial movement along actin filaments has been widely described, there are still many unanswered questions in this complex process that need to be further explored.

## Conclusion and Prospects

Disruption of mitochondrial function leads to abnormal cell fate and a range of diseases. In this review, we summarize the roles of mitochondrial dynamics and function in diseases such as metabolic diseases, CVDs, neurodegenerative diseases, and cancer, including mitochondrial fusion and fission, mitochondrial autophagy and transport, and mitochondrial function, and describe the roles played by a series of key proteins that provide new insights into targeting mitochondria to regulate disease.

In addition, mitochondria are highly plastic and dynamic organelles that regulate their morphology and function in response to a variety of stimuli both inside and outside the cells [5]. In this review, we summarize the effects of intra- and extracellular biochemical and mechanical cues on mitochondrial dynamics, function, and transfer, and highlight the specific molecular mechanisms by which intra- and extracellular mechanical cues regulate mitochondrial morphology and function and transfer. Alterations in these mechanical signals arise with diseases such as aging, fibrosis, and cancer. Altered mechanical signals can in turn be transmitted to mitochondria via intra- and extracellular mechanotransduction molecules, thereby altering their morphology and function, ultimately leading to malignant cellular behaviors. Therefore, studying the results of tissue and cell mechanics associated with common diseases may be a new way to treat them.

Although we have identified many mechano-regulated phenomena of mitochondrial dynamics and function, the underlying mechanotransduction mechanisms remain to be refined. Ca^2+^ are usually thought to enter mitochondria through the mitochondrial calcium uniporter (MCU) on the mitochondrial membrane and regulate its function [141]. Recent studies have shown that connexins are also localized to mitochondria, particularly in the heart, where they regulate mitochondrial ion homeostasis (K^+^, Ca^2+^) by forming mitochondrial hemichannels [142]. Whether mitochondrial connexin 43 responds to Piezo1/TRPV4 remains to be investigated. In addition, most of the experiments did not investigate the key mechanosensitive proteins and specific signaling pathways, while others were limited to cell lines and experimental conditions, and the conclusions obtained could not be applied to other cell lines. For example, normal cells and tumor cells show different mitochondrial morphology and function under similar matrix stiffness conditions, and the specific regulatory substrates behind this need to be investigated. However, what we can affirm is the indispensable and important role of mitochondrial dynamics in mechanotransduction [123].

In addition, most in vitro studies are established in 2D conditions, whereas in vivo cells are in more complex environments and are subjected to multifaceted mechanical stimuli; thus, further optimization of in vitro models to reflect the true state of the cells under physiological or pathological conditions is needed in the future. For example, in vivo cells often move in irregular channels, and we would like to understand how the mechanical stimuli to which the cells are subjected change during such complex movements, and how the cells regulate mitochondrial dynamics and function to adapt to the environmental stimuli. Therefore, we need to establish a reasonable and perfect in vitro model for the study. Moreover, when performing in vivo experiments, we hope to develop an in vivo real-time mitochondrial tracer model in the future to replace the traditional immunohistochemistry to better observe the changes of mitochondrial dynamics. Last but most importantly, as our research continues, we need to consider how to turn these mechanosignaling molecules into powerful targets for the treatment of various diseases.
